# Selective Fetal Growth Restriction in Dichorionic Twin Pregnancies: Diagnosis, Natural History, and Perinatal Outcome

**DOI:** 10.3390/jcm9051404

**Published:** 2020-05-09

**Authors:** Nikolaos Antonakopoulos, Petra Pateisky, Becky Liu, Erkan Kalafat, Baskaran Thilaganathan, Asma Khalil

**Affiliations:** 1 Fetal Medicine Unit, St George’s University Hospitals, Blackshaw Road, London SW17 0QT, UK; n.antonakopoulos@yahoo.gr (N.A.); petra.pateisky@meduniwien.ac.at (P.P.); bexliu@doctors.org.uk (B.L.); basky@pobox.com (B.T.); 2 3rd Department of Obstetrics Gynaecology and Feto-Maternal Medicine, University of Athens Medical School, Attikon Hospital & Gynecology Obstetrics and Perinatal Medicine Unit, Evgenideio Hospital, 11528 Athens, Greece; 3 Division of Obstetrics and Feto-Maternal Medicine, Department of Obstetrics and Gynaecology, Medical University of Vienna, 1090 Vienna, Austria; 4 Department of Statistics, Faculty of Arts and Sciences, Middle East Technical University, Ankara 06800, Turkey; mail@erkankalafat.com; 5 Vascular Biology Research Centre, Molecular and Clinical Sciences Research Institute, St George’s University of London, Cranmer Terrace, London SW17 0RE, UK

**Keywords:** DCDA twins, selective fetal growth restriction, morbidity and mortality, natural history, diagnostic criteria

## Abstract

This study aims to evaluate the natural history, disease progression, and outcomes in dichorionic twins with selective fetal growth restriction (sFGR) according to different diagnostic criteria and time of onset. Dichorionic twins seen from the first trimester were included. sFGR was classified according to the Delphi consensus, and was compared to the outcomes of those classified by the International Society of Ultrasound in Obstetrics and Gynecology (ISUOG) diagnostic criteria. Early sFGR occurred before 32-weeks, and late sFGR after 32-weeks. Disease progression, neonatal outcomes such as gestation at delivery, birthweight, neonatal unit (NNU) admission, and morbidities were compared. One-hundred twenty-three of 1053 dichorionic twins had sFGR, where 8.4% were classified as early sFGR, and 3.3% were late sFGR. Disease progression was seen in 36%, with a longer progression time (5 vs. 1 week) and higher progression rate (40% vs. 26%) in early sFGR. Perinatal death was significantly higher in the sFGR than the non-sFGR group (24 vs. 16 per 1000 births, *p* = 0.018), and those with early sFGR had more NNU admissions than late sFGR (*p* = 0.005). The ISUOG diagnostic criteria yielded a higher number of sFGR than the Delphi criteria, but similar outcomes. sFGR have worse perinatal outcomes, with early onset being more prevalent. Use of the Delphi diagnostic criteria can reduce over-diagnosis of sFGR and avoid unnecessary intervention.

## 1. Introduction

Twin pregnancies carry a higher risk of prenatal complications, such as selective fetal growth restriction (sFGR), preterm birth, perinatal morbidity, and mortality [1]. sFGR is seen less commonly in dichorionic (DC) than monochorionic (MC) twin pregnancies, with a reported prevalence of 10.5% compared to 19.7%, respectively [2]. DC twin pregnancies are believed to have a lower perinatal mortality rate than MC twins (33 per 1000 vs. 75 per 1000) [3], as well as a lower rate of neurological co-morbidities [4]. The recommended management for sFGR in DC twin pregnancies is the same as that of growth restricted singletons [1,5]. However, the evidence available to support this is scarce [6], and most of the literature on this subject largely focuses on MC twins. 

sFGR in MC twins is thought to be caused by an unequal sharing of the placenta and distribution of blood through placental anastomoses [7], whereas in DC twins, from placental insufficiency in one of the placentas [4]–explaining the higher incidence of pre-eclampsia in DC than MC twins with sFGR [8]. Other causes can include congenital infections, or discordant anomalies, which can be excluded through detailed ultrasound assessment, maternal serology and invasive prenatal testing [5]. Recent evidence has suggested that the natural history of sFGR in DC twins may not be so similar to that of singletons. Vanlieferinghen et al have found that the interval from development of umbilical artery Doppler abnormalities to birth was significantly longer in growth restricted DC twins than in growth restricted singletons [9].

The classification of sFGR has shown significant variation, making evaluations and comparisons of prevalence and outcomes of this pathology difficult. The International Society of Ultrasound in Obstetrics and Gynecology (ISUOG) defines sFGR in DC twins as an estimated fetal weight (EFW) <10^th^ centile [5,10]. A recent consensus, using the Delphi procedure, has focused on achieving uniform diagnostic criteria and reporting parameters in twin pregnancies with sFGR. This classified sFGR in DC twins as either the EFW of one twin <3^rd^ centile, or when two of the following parameters were met-EFW of one twin <10^th^ centile, EFW discordance >25%, or umbilical artery pulsatility index (UA PI) >95^th^ centile of the smaller twin [10]. The main aim of this study is to assess the natural history and perinatal outcomes of sFGR in DC twin pregnancies, according to the time of onset and the different reported diagnostic criteria.

## 2. Experimental Section

This was a longitudinal cohort study of all dichorionic diamniotic (DCDA) twin pregnancies that had their routine antenatal care at the Fetal Medicine Unit at St. George’s Hospital, University of London. The pregnancies were identified retrospectively by searching the ultrasound database (ViewPoint version 5.6.26.148, ViewPoint Bildverarbeitung GMBH, Wessling, Germany). All DCDA twin pregnancies with confirmed chorionicity at 11–14 weeks of gestation, who underwent routine scans at our Fetal Medicine Unit from January 2000 until January 2019 were included in the study. Dichorionicity was diagnosed using the presence of the lambda-sign in the inter-twin membrane at the site of membrane insertion into the placenta, during the first trimester ultrasound scan between 11 and 14 weeks. Gestational age (GA) was determined according to the crown rump length (CRL) of the larger twin in spontaneous pregnancies, and according to the date of oocyte retrieval or embryonic age from fertilization in cases of pregnancies conceived via in vitro fertilization (IVF). The patients had four weekly scans following the anomaly scan, and 1–2 weekly scans after the diagnosis of sFGR, or more frequently if clinically indicated as per the fetal umbilical artery Doppler findings. 

Pregnancies diagnosed with sFGR underwent testing modalities in the form of a detailed anomaly scan, Doppler (umbilical artery, middle cerebral artery, and ductus venosus) assessment, TORCH screen, and karyotyping if they wished to exclude genetic syndromes and aneuploidies. Pregnancies complicated by major fetal structural anomalies, aneuploidy, genetic syndromes or lost to follow-up were excluded from the analysis. We also excluded the pregnancies where both fetuses were growth restricted. The pregnancy outcomes were obtained from the maternity database and the neonatal records. Those with positive TORCH results were counseled and managed accordingly. The pregnancies were managed according to the following protocol: expectant management prior to 30 weeks’ gestation; elective birth if abnormal ductus venosus (DV) Doppler at or beyond 30 weeks after a course of steroids; elective birth if reversed end-diastolic flow (EDF) in the umbilical artery (UA) at or beyond 31–32 weeks after a course of steroids, elective birth if absent EDF in the UA at or beyond 32–33 weeks after a course of steroids; elective birth if the UA pulsatility index (PI) is at or above the 95^th^ centile (even with positive EDF) at or beyond 34 weeks after a course of steroids; elective birth if middle cerebral artery (MCA) PI <5^th^ centile at or beyond 36 weeks; and deliver all those with normal fetal Dopplers at or beyond 37 weeks. Pregnant women who underwent planned delivery before 34 weeks’ gestation underwent a Caesarean section, as did those who had maternal reasons for Caesarean section who delivered after 34 weeks.

The inter-twin discordance was calculated as the EFW difference divided by the EFW of the larger twin multiplied by 100. The EFW prior to 20 weeks was derived by the formula by Warsof et al., and at 20 weeks or beyond it was derived by the formula of Hadlock et al [11,12]. Growth was evaluated using the STORK twin growth charts [13]. Birthweight percentiles were calculated using twin chorionicity specific reference standards reported by Ananth et al [14]. Based on the recently published consensus on the diagnostic criteria for sFGR in twin pregnancies [10], one solitary parameter irrespective of chorionicity (EFW of one twin < 3^rd^ centile), or two of the three contributory criteria (EFW of one twin < 10^th^ centile, EFW discordance ≥ 25%, or UA PI of the smaller twin > 95^th^ centile) were used to diagnose sFGR. A pregnancy was considered to be affected by sFGR if it fulfilled the criteria in two or more scans in order to avoid over-diagnosis taking into account the limitations of ultrasound assessment of the EFW in twin pregnancies.

In line with a recent Delphi consensus on fetal growth restriction (FGR) in singleton pregnancies [15], we defined early sFGR as sFGR occurring before 32 weeks and late sFGR as after 32 weeks’ gestation. This difference in classification from MC twins (early sFGR before 24 weeks and late sFGR after 24 weeks) was due to the fact that DC twins with sFGR would unlikely undergo the same interventions as MC twins (e.g., fetoscopic laser or bipolar cord occlusion), and are often managed similarly to singleton pregnancies. Doppler assessment of the UA of both twins and the classification of EDF as normal, absent, or reversed was carried out in all cases, at each ultrasound assessment. Miscarriage was defined as the death of at least one twin up to 20 weeks’ gestation and intrauterine demise (IUD) after 20 weeks. Neonatal death (NND) was defined as the death of at least one newborn up to 28 days of life. Perinatal death (PND) was defined as the sum of IUD and NND. 

To assess disease progression of the sFGR twin, we calculated the time interval between the gestational age at the first ultrasound scan at diagnosis, and the scan displaying the following worsening fetal parameters: increase in the UA PI from normal to above the 95^th^ centile, or EDF changes from positive to absent, or from absent to reversed EDF, or the presence of an absent or reversed a-wave in the ductus venosus.

### Statistical Analysis

Continuous variables were presented as either median and interquartile range or mean and standard deviation according to distribution characteristics. Normality assumptions were tested with Shapiro-Wilk test. Continuous variables were compared using either t-test or Wilcoxon rank-sum test depending on the distribution characteristics. Categorical variables were compared using chi-squared test or Fisher’s exact test where appropriate. For comparison using fetus level data, generalized estimating equations were used to account for inter-twin dependency structure. *p* values below 0.05 were considered statistically significant. All statistical analyses were performed using R for statistical computing software and using geepack package [16]. 

## 3. Results

### 3.1. Key Findings

#### 3.1.1. Study Population

**Figure 1:**
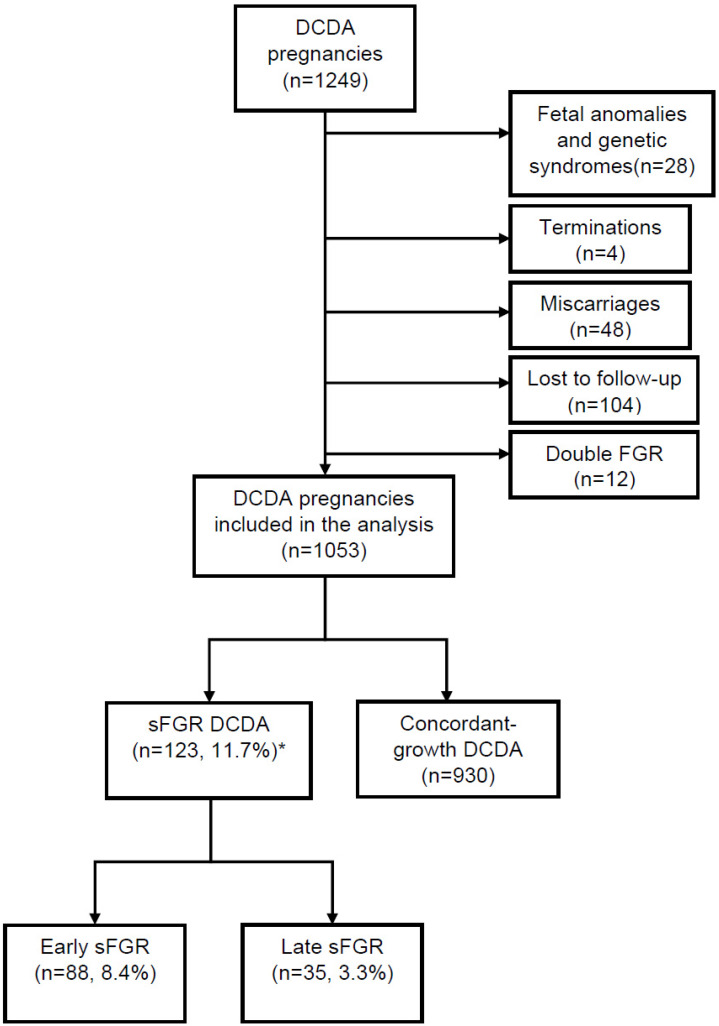
Flow diagram representing the whole study population of dichorionic diamniotic twin pregnancies (*n* = 1249). DCDA: dichorionic diamniotic, sFGR: selective fetal growth restriction. * Incidence of sFGR classified according to Delphi diagnostic criteria.

Our study cohort included 1249 DC twin pregnancies. Figure 1 shows a flowchart of the initially 1249 screened DCDA twin pregnancies and the reasons for the exclusion of 196 DCDA twin pregnancies from further analysis. Twenty-eight pregnancies were excluded due to fetal anomalies, 12 pregnancies had FGR in both babies, 4 pregnancies were terminated, 48 pregnancies miscarried, and 104 were lost to follow-up. A total of 1053 DC twin pregnancies were included in our final analysis. There were no significant differences in maternal age (*p* = 0.409), body mass index (BMI) (*p* = 0.231), or mode of conception (*p* = 0.333) between sFGR and normal groups (Table 1). There were more women of Asian origin (21.1% vs. 12.2%, *p* = 0.032) and less women of Afro-Caribbean origin (8.9% vs. 14.1%, *p* = 0.032) in the sFGR group than the group without sFGR.

#### 3.1.2. Incidence of sFGR According to Diagnostic Criteria and Gestational Age at Diagnosis

The incidence of sFGR according to the different diagnostic criteria and gestational age at diagnosis is shown in Table 2. According to the Delphi diagnostic criteria, 11.7% of the DC twin pregnancies were classified as sFGR; 88 (8.4%) were early onset (<32 weeks), and 35 (3.3%) late-onset (≥32 weeks) sFGR. Conversely, using the classification of early and late sFGR according to MC twins (before and after 24 weeks), 34 (3.2%) would be classified as early onset sFGR, and 89 (8.5%) late-onset sFGR. The criteria with the least incidence of sFGR was EFW discordance of 25% plus umbilical artery PI of the smaller twin >95^th^ centile (*n* = 19, 1.8%). GA at diagnosis were similar in all criteria used, and regardless of diagnostic criteria, early sFGR was more prevalent than late sFGR.

#### 3.1.3. Disease Progression, Clinical Deterioration and Perinatal Outcomes

In those twin pregnancies diagnosed with sFGR according to the Delphi criteria, 44 (35.8%) showed progression or clinical deterioration-39.8% of the early sFGR, compared to 25.7% of the late sFGR. The interval between diagnosis and disease progression was 5 weeks in early sFGR, and one week in the late sFGR group, with an average of 4 weeks in all the sFGR pregnancies (Table 3). 

Stillbirth, neonatal and perinatal death were more common in the early sFGR group (11 per 1000 births, *p* < 0.001, 23 per 1000 births, *p* < 0.001, and 34 per 1000 births, *p* = 0.178 respectively). We had no pregnancies complicated by IUD, NND or PND in the late sFGR group. There were no significant differences in intrauterine demise (*p* = 0.664) or neonatal death (*p* = 0.464) between the sFGR and non-sFGR groups; however, the perinatal death in the sFGR group was significantly higher than the non-sFGR group (*p* = 0.018) (Table 4).

Pregnancies complicated by early sFGR were delivered earlier than late sFGR (34.0 vs. 35.0 weeks, *p* < 0.083), and the birthweight was lower (2044g vs. 2128g, *p* = 0.011 in the larger twin, and 1529g vs. 1740g, *p* = 0.074 in the smaller twin); however, these results, with the exception of the birth weight of the larger twin, were not statistically significant. NNU admissions were significantly higher in those with early sFGR than late sFGR (*p* = 0.005). 

**Figure 2:**
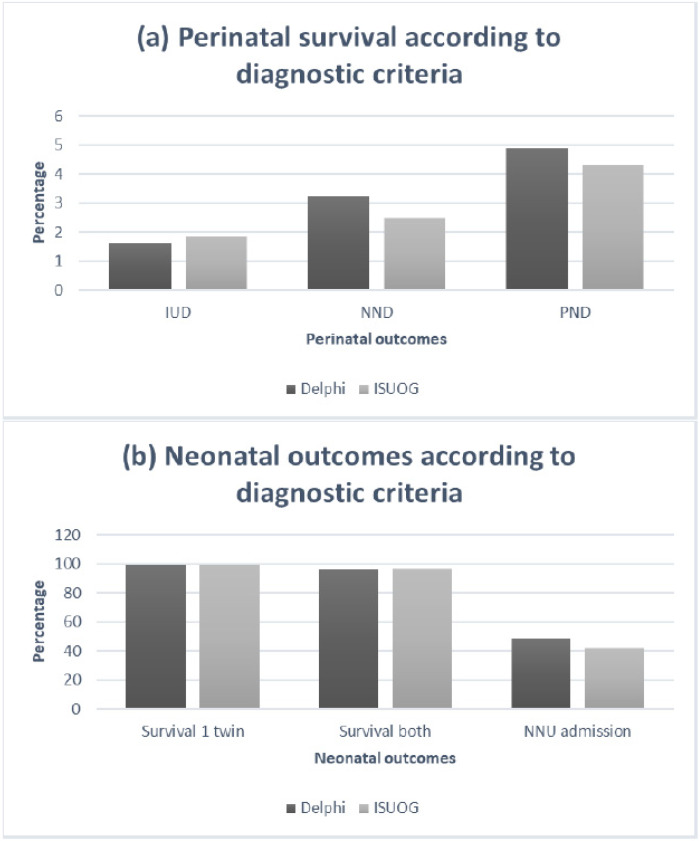
Perinatal and neonatal outcomes of sFGR DCDA twins according to Delphi and ISUOG diagnostic criteria: (**a**) Bar chart demonstrating the incidence of IUD (intrauterine demise), NND (neonatal death), and PND (perinatal death) in the Delphi and ISUOG criteria groups; (**b**) Bar chart demonstrating the incidence of neonatal outcomes (survival of one twin, survival of both twins, and neonatal unit (NNU) admissions) according to Delphi and ISUOG criteria groups.

We also performed an analysis of pregnancy outcomes for sFGR diagnosed according to the ISUOG criteria (Table 5). The differences in outcomes compared to the no sFGR group, and the comparisons between the early and late sFGR, were similar to those found diagnosed as sFGR by the Delphi diagnostic criteria only. The outcomes compared with the Delphi diagnosed cases are demonstrated in Figure 2. GA at delivery (35 vs. 34 weeks) and birthweight (1720 vs. 1660g in smaller twin, and 2195 vs. 2107g in larger twin), although higher, were still significantly lower than the non-sFGR group (*p* < 0.001 in both). The IUD (9 per 1000 births vs. 5 per 1000 birth) and PND (22 per 1000 births vs. 16 per 1000 birth) were not significantly different in the sFGR versus non-sFGR groups (*p* > 0.05). Both outcomes were more common in the early sFGR (13 per 1000 births and 30 per 1000 births, respectively). Of the pregnancies classified as sFGR according to ISUOG criteria, but did not meet the Delphi criteria (*n* = 39), only one pregnancy suffered an IUD, with no cases of NND. This occurred in the larger twin at 28 weeks, and no obvious cause was found for the demise. 

## 4. Discussion

### 4.1. Summary of the Study Findings

According to ISUOG diagnostic criteria (EFW of one twin < 10^th^ centile), the incidence of sFGR in this population of unselected dichorionic twin pregnancies was 15.4%. The incidence varied according to the diagnostic criteria used, with lower incidence when using the recently published Delphi diagnostic criteria (11.7%). In pregnancies diagnosed with sFGR, the GA at delivery was significantly earlier, and the NNU admission higher, than those without sFGR. The overall prevalence of early sFGR (72%) was significantly higher than late sFGR (28%). The rate of neonatal unit admission was significantly higher in the early sFGR group. These pregnancies also showed a higher progression rate than the late sFGR group, but a longer interval between diagnosis to disease progression.

### 4.2. Clinical and Research Implications

The ISUOG diagnostic criteria classified the highest number of twins as sFGR, and the lowest number was classified according to the Delphi criteria of inter-twin discordance >25% and UA PI >95^th^ centile. The latter category may therefore reflect the most severe form of sFGR, whereas the former criteria may diagnose healthy small twins as sFGR. The one case of IUD in the cohort classified as sFGR according to the ISUOG but not the Delphi criteria was unlikely related to sFGR, as it occurred in the larger twin, and demonstrates the likelihood that this group of pregnancies may in fact be healthy small twins. Twins are known to have lower than average birth weights, as well as slowed growth in the third trimester [17]. Despite this, singleton growth charts are still routinely used in twin pregnancies, leading to an over-diagnosis of small for gestational age (SGA), with no increased risk of perinatal death in DC twins compared to singletons [18]. We published twin specific growth ranges through the analysis of a large cohort of twin pregnancies, which is now routinely used in our unit, and also for the calculations in our study [13,19,20]. In the latter study by Stirrup et al., we found that DC twins followed a similar growth trajectory to singletons until 30 weeks, followed by a relative reduction in growth velocity thereafter [13]. Therefore, by solely using centile-based criteria (e.g., EFW < 10^th^ centile) to diagnose sFGR, significant variation in sFGR prevalence can be introduced depending on the growth chart used. The inevitable consequences of the latter are over-diagnosis of sFGR and unnecessary iatrogenic early delivery of appropriate for gestational age (AGA) twins.

Management of sFGR in twin pregnancies poses a clinical conundrum. It is recognised that growth discordance, particularly when one is growth restricted, can carry an increased risk of perinatal death [21]. Expectant management can result in single fetal demise in the growth restricted twin, which can in turn lead to preterm delivery in 54%, and carries a 2% risk of neurological damage, and 3% risk of co-twin demise in the live twin [22]. Elective premature birth, however, carries significant risks of prematurity in the AGA twin. Selective fetal reduction has a limited role in DC twins, due to the risk of preterm birth, but has been reported to be used in cases of severe preterm pre-eclampsia [23].

Our results illustrate the importance of using standard diagnostic criteria of sFGR and could explain the large variation among the published studies. Recent international efforts have also focused on standardizing the outcomes reported in twin studies [24,25]. One future research priority, which has been identified by the global twins and multiples priority setting partnership, is how we can assess the growth of twin infants after birth and how to ensure that they follow a satisfactory growth trajectory [26]. Despite the fact that the EFW reference ranges are representative of the whole population, the traditional approach of deriving birth weight charts has been criticised, as a large proportion of babies born preterm could result from pathological pregnancies. This is a significant concern in twin pregnancies in view of the high rate of preterm birth.

### 4.3. Interpretation of Study Findings and Comparison with Existing Literature

As the existing literature on sFGR in DC twin pregnancies is scarce, and they are managed in a similar fashion to singleton pregnancies, we have adopted an approach to extrapolate from singletons with FGR. Vanlieferinghen et al. found that the time from the first abnormal umbilical artery Doppler finding to the time of delivery was significantly longer in growth restricted DC twins than in singletons (53 vs. 16 days), and that twins with sFGR were delivered later than singletons, with no difference in outcome [9]. This could be due to clinician bias, in order to delay delivery for the sake of the AGA twin, or could reflect a slower disease progression in twins compared to singletons. Comparing our findings to their singleton data, a mean interval from diagnosis to delivery was 21 days in their singleton group, and in our cohort, the mean interval from diagnosis to disease progression (i.e., Doppler deterioration) was four weeks (28 days). Our mean birthweight was 1801g in the sFGR group, and their singletons had a median birthweight of 1380g. The gestation at delivery in our sFGR group was comparable to their singleton group (33.99 vs. 33.6 weeks). The TRUFFLE (Trial of Randomized Umbilical and Fetal FLow in Europe) study analysed perinatal outcomes in a cohort of severe early onset growth restricted singleton pregnancies. The average gestation at delivery was 30 + 5 weeks, with a diagnosis to delivery time of 8 days, and a mean birth weight of 1013g [27]. However, their diagnostic criteria used abdominal circumference <10^th^ centile, and UA PI >95^th^ centile, which differs from our study criteria.

The lower progression rate and better outcomes in the late sFGR group in this study may be explained, by the fact that they were electively delivered shortly following diagnosis, due to their later gestations. The lack of significant difference in the intrauterine or neonatal deaths in the DC twin pregnancies complicated by sFGR could also be explained by intervention bias. As these pregnancies were monitored closely and delivered early, therefore, reducing the risk of intrauterine demise of the smaller twin. However, when combined, the rate of perinatal mortality was significantly higher in the sFGR group, which may be more highly contributed by the number of neonatal deaths, secondary to the lower gestation at delivery and lower birthweights. In light of these findings, it may be reasonable to suggest that management can be more conservative in DC twin pregnancies, and unnecessary preterm delivery may be reduced with continued close ultrasound observation.

We have recently published a study describing the natural history of sFGR in monochorionic twin pregnancies according to the recent diagnostic criteria [28]. The incidence of early sFGR was 4.9%, while that of late sFGR was 3.8%, according to ISUOG diagnostic criteria. When applying the various diagnostic criteria, the incidence of early sFGR varied from 1.7% to 9.1% and late sFGR from 1.1% to 5.9%. It is important though to point out that we used a different cut-off (<24 weeks’ gestation) to define early vs. late-onset sFGR taking into account viability and gestational age threshold of active fetal intervention in these pregnancies.

### 4.4. Strengths and Limitations

Our study reports the prevalence of early and late sFGR in DC twins according to different diagnostic criteria, the natural history of the disease, and perinatal outcomes, in a large cohort of twin pregnancies. The main limitation is its retrospective nature, and that we did not adjust for the background patient demographics. However, it is unlikely to impact the study outcomes. The link of a priori risk factors and sFGR in DC twin pregnancies is not well established and the published literature reports controversial findings [29,30,31,32,33]. The lack of significant differences in still birth and neonatal deaths in sFGR and non-sFGR, as well as between the early and late sFGR groups should be interpreted with caution as the number of pregnancies in these analyses might be too small. For future research, it would be beneficial to compare these outcomes and disease progression with singleton pregnancies using the same diagnostic criteria, in order to ascertain whether there is a true difference in the natural history of the disease. As the clinicians were not blinded to the ultrasound findings, the potential risk of intervention bias is very likely.

Due to the number of patients who were lost to follow-up, and the patients who delivered prior to the availability of an electronic neonatal database, we were unable to adequately retrieve neonatal morbidity data to allow for reliable analysis, which would have provided additional valuable information in the comparison of the diagnostic criteria. Furthermore, the retrospective study design poses a risk of selection and classification bias, and this together with patients who were lost to follow-up can make accurate comparisons difficult to make. Therefore, these outcomes should also be interpreted with caution. Future prospective studies using population-based registries, which extends to long-term follow-up for these twins will be beneficial in providing a more precise assessment of development and growth trajectories, as well as developmental information.

## 5. Conclusions

The incidence of sFGR in DC twin pregnancies varies according to the diagnostic criteria used. The ISUOG diagnostic criteria, compared to the Delphi diagnostic criteria, may lead to early iatrogenic delivery and unnecessary parental anxiety. Early sFGR is significantly more prevalent than late sFGR, and has a worse perinatal outcome. The overall progression of disease is seen in a third of these pregnancies, with a longer time of progression than reported in singleton pregnancies. The use of twin specific growth charts and the Delphi diagnostic criteria may reduce the diagnosis of sFGR, without a significant effect on perinatal mortality, and possibly reducing perinatal morbidities through avoiding unnecessary preterm deliveries. Further prospective studies extending to long-term follow-up of these twins will help to provide more accurate comparisons and developmental assessments.

## Figures and Tables

**Table 1:** Baseline characteristics of the dichorionic diamniotic (DCDA) twin pregnancies, according to whether the pregnancy was complicated by selective fetal growth restriction (sFGR) or not.

	sFGR * (*n* = 123)	No sFGR (*n* = 930)	*p*-Value
**Maternal age in years (mean ± SD)**	34.0 (29.0–36.0)	34.0 (30.0–36.0)	0.409
**Maternal body mass index in Kg/m^2^ (mean ± SD)**	24.0 (21.0–27.0)	24.0 (22.0–27.4)	0.231
**Mode of conception**			
**Spontaneous conception,** ***n*** **(%)**	83 (67.5)	586 (63.0)	0.333
**IVF,** ***n*** **(%)**	40 (32.5)	344 (37.0)	
**Ethnicity,** ***n*** **(%)**			
**White**	79 (64.2)	639 (68.7)	0.032
**Black**	11 (8.9)	131 (14.1)	
**Asian**	26 (21.1)	113 (12.2)	
**Other**	7 (5.7)	47 (5.1)	

* Classified according to the Delphi diagnostic criteria.

**Table 2:** Incidence of selective fetal growth restriction (sFGR) in dichorionic twin pregnancies according to the various proposed diagnostic criteria as stated by a consensus reached through a Delphi process (Khalil A 2019) and ISUOG twin guideline, as well the gestational age at diagnosis. Data are shown as number (%) or median (IQR).

Diagnostic Criteria	Incidence of sFGR	Incidence of Early sFGR	GA Diagnosis Early sFGR (wks)	Incidence of Late sFGR	GA Diagnosis Late sFGR (wks)
**Delphi criteria A** EFW < 3^rd^ centile of one twin	95 (9.0)	80 (7.6)	27.0 (22.0–28.0)	15 (1.4)	34.0 (33.0–35.0)
**Delphi criteria B** EFW < 10^th^ centile of one twin + inter-twin EFW discordance ≥ 25%	49 (4.7)	39 (3.7)	26.0 (22.0–28.0)	10 (1.0)	34.0 (32.0–35.0)
**Delphi criteria C** EFW < 10^th^ centile of one twin + umbilical artery PI > 95^th^ centile	60 (5.7)	36 (3.4)	28.0 (22.0–28.0)	24 (2.3)	33.5 (32.7–34.0)
**Delphi criteria D** Inter-twin EFW discordance ≥ 25% + umbilical artery PI > 95^th^ centile	19 (1.8)	16 (1.5)	25.5 (22.0–28.0)	3 (0.3)	32.0 (32.0–32.0)
**ISUOG criteria** EFW < 10th centile of one twin	162 (15.4)	116 (11.0)	27.0 (22.0–28.0)	46 (4.4)	34.0 (33.0–35.0)

ISUOG: The International Society of Ultrasound in Obstetrics and Gynecology; GA: gestational age; EFW: estimated fetal weight; PI: pulsatility index; *n*: number of patients.

**Table 3:** Disease progression in dichorionic twin pregnancies complicated by selective fetal growth restriction (sFGR) according to the gestational age at diagnosis.

	sFGR (*n* = 123)	Early Onset sFGR (*n* = 88)	Late-Onset sFGR (*n* = 35)
Progression, *n* (%)	44 (35.8)	35 (39.8)	9 (25.7)
Interval between diagnosis and progression in weeks, median (IQR)	4 (2–7)	5 (1–7.5)	1 (1–1)
Stable, *n* (%)	79 (64.2)	53 (60.2)	26 (74.3)

**Table 4:** Perinatal outcomes in dichorionic twin pregnancies according to whether they were complicated by sFGR or not according to the Delphi diagnostic criteria and stratified according to the gestational age at diagnosis. Data are shown as number (%) or median (IQR).

	sFGR (*n* = 123)	No sFGR (*n* = 930)	*p*-Value	Early sFGR (*n* = 88)	Late sFGR (*n* = 35)	*p*-Value
Gestation at birth (weeks)	34.0 (33.0–36.0)	37.0 (35.0–37.0)	<0.001	34.0 (31.8–36.0)	35.0 (33.5–36.0)	0.083
Birth weight (g), larger baby	2107 (1771–2339)	2640 (2345–2920)	<0.001	2044 (1656–2328)	2128 (1985–2377)	0.011
Birth weight (g), smaller baby	1660 (1192–1846)	2362 (2080–2601)	<0.001	1529 (1130–1815)	1740 (1588–1895)	0.074
Birth weight centile, larger baby	36.0 (18.7–55.1)	63.5 (43.4–82.1)	<0.001	39.3 (11.6–80.5)	20.7 (1.2–72.3)	0.062
Birth weight centile, smaller baby	3.2 (1.1–7.8)	34.3 (14.4–52.7)	<0.001	5.6 (0.5–12.9)	4.6 (0.3–13.6)	0.295
Intrauterine demise (per 1000 total birth †)	2 (8)	10 (5)	0.644 ‡	2 (11)	0 (0)	<0.001 ‡
Neonatal death (per 1000 live birth †)	4 (16)	19 (10)	0.464 ‡	4 (23)	0 (0)	<0.001 ‡
Perinatal death (per 1000 total birth †)	6 (24)	29 (16)	0.018 ‡	6 (34)	0 (0)	0.178 ‡
Survival of at least one twin	122 (99.2)	925 (99.5)	0.703	87 (98.9)	35 (100.0)	0.999
Survival of both twins	118 (95.9)	896 (96.3)	0.821	83 (94.3)	35 (100.0)	0.350
Neonatal unit admission *	133 (54.5)	389 (21.2)	<0.001 ‡	105 (60.3)	28 (40.0)	0.005 ‡

* Denominator excludes fetuses complicated by intrauterine death or missing neonatal admission outcome † Rounded to nearest whole number ‡ Generalized estimating equation model results.

**Table 5:** Perinatal outcomes in dichorionic twin pregnancies according to whether they were complicated by sFGR or not according to the ISUOG diagnostic criteria in the whole cohort and stratified according to the gestational age at diagnosis. Data are shown as number (%) or median (IQR).

	sFGR (*n* = 162)	No sFGR (*n* = 891)	*p*-Value	Early sFGR (*n* = 116)	Late sFGR (*n* = 46)	*p*-Value
Gestation at birth (wks)	35.0 (33.0–37.0)	37.0 (35.0–37.0)	<0.001	35.0 (32.0–37.0)	35.0 (34.0–36.7)	0.131
Birth weight (g), larger baby	2195 (1856–3450)	2660 (2350–2950)	<0.001	2164 (1738–2447)	2222 (2024–2530)	0.062
Birth weight (g), smaller baby	1720 (1400–1920)	2390 (2105–2615)	<0.001	1680 (1173–1910)	1747 (1645–1958)	0.062
Birth weight centile, larger baby	36.2 (21.0–54.3)	64.4 (44.8–82.6)	<0.001	34.6 (22.2–51.7)	38.0 (18.9–58.4)	0.568
Birth weight centile, smaller baby	3.9 (1.5–8.0)	35.8 (18.9–55.3)	<0.001	3.5 (1.1–7.3)	4.8 (1.8–9.2)	0.194
Intrauterine demise (per 1000 total birth †)	3 (9)	9 (5)	0.370 ‡	3 (13)	0 (0)	<0.001 ‡
Neonatal death (per 1000 live birth †)	4 (12)	19 (11)	0.758 ‡	4 (17)	0 (0)	<0.001 ‡
Perinatal death (per 1000 total birth †)	7 (22)	28 (16)	0.069 ‡	7 (30)	0 (0)	0.150 ‡
Survival of at least one twin	161 (99.4)	888 (99.7)	0.593	115 (99.1)	46 (100.0)	0.999
Survival of both twins	156 (96.3)	866 (97.2)	0.534	110 (94.8)	46 (100.0)	0.266
Neonatal unit admission *	155 (48.4)	367 (20.8)	<0.001 ‡	121 (53.1)	34 (37.0)	0.009 ‡

* Denominator excludes fetuses complicated by intrauterine death or missing neonatal admission outcome † Rounded to nearest whole number ‡ Generalized estimating equation model results.
